# United States Medical Library

**Published:** 1878-01

**Authors:** 


### United States Medical Library.

The British and Foreign Medico-Chirugical Review, says: "We warmly congratulate the profession of the United States on their good fortune in possessing so able a bibliographer as Dr. Billings, who has the intellect to appreciate the importance of a great public medical library, and the enthusiasm and diligence sufficient to overcome the endless difficulties met with in the formation and management of such a collection. Surely his efforts and his great success will have a reflex action on European, especially on English, librarians. Nowhere are there such numerous opportunities for buying rare and curious books and manuscripts, as in the London auction-rooms, yet no use seems to be made of them by any of the London medical libraries at the present time."

